# Brucellosis in livestock and wildlife: zoonotic diseases without pandemic potential in need of innovative one health approaches

**DOI:** 10.1186/s13690-017-0207-7

**Published:** 2017-09-11

**Authors:** Jacques Godfroid

**Affiliations:** Department of Arctic and Marine Biology, Research Group of Arctic Infection Biology, University of Tromsø - the Arctic University of Norway, Faculty of Biosciences, Fisheries and Economics, Langnes, Postbox 6050, 9037 Tromsø, Norway

## Abstract

Human brucellosis remains the commonest zoonotic disease worldwide with more than 500 000 new cases annually. Understanding the biology of *Brucella* infections and the transmission patterns at the wildlife/livestock/human interface is of paramount importance before implementing any brucellosis control or eradication program in animals, even more so should interventions be justified within One Health. In addition to calling for transdisciplinary collaboration, One Health formally aims to conserve the environment and to promote the well-being of animals. In this opinion paper, the One Health approach of brucellosis is reviewed in the industrialized and the low and middle income countries, highlighting pitfalls and shortcomings of serological studies and discussing the role of urban and peri-urban farming for the re-emergence of brucellosis in the developing world. The role of wildlife as a potential reservoir is highlighted and different management strategies are discussed. Lastly, beyond its role in the control of brucellosis, the ethical dimension of culling wildlife to control disease emergence or spill-back of infections in livestock is discussed. Core transdisciplinary competencies such as values and ethics are critically important in guiding the development of One Health curricula and in continuing professional education, as they describe the knowledge, skills, and attitudes required to be effective. A conceptual framework needs to be developed from inception to knowledge translation. Importantly, transdisciplinary competencies should be developed as an adjunct to discipline-specific areas of expertise, not as a replacement. A profound understanding of the biology of infectious agents is and will always remain a pre-requisite for any sound One Health approach.

## Introduction

In his foreword to the book “People, Pathogens, and Our Planet” – Volume 1: Towards a One Health Approach for Controlling Zoonotic Diseases, Juergen Voegele, Director of Agriculture and Rural Development at the World Bank wrote the following: “A global surveillance and control system that is established primarily for emerging infectious zoonotic diseases with pandemic potential can be readily improvised to address the endemic diseases that are a priority in many developing countries, few of which have the capacity or resources necessary to monitor or control them effectively” [1].

A global surveillance and control system cannot (and should not!) be improvised for endemic zoonotic diseases. Indeed, endemic and epidemic diseases are in essence different and do require tailored approaches. For example, the implementation of an early detection/warning system, which is the first stage of any control program of epidemicc diseases is meaningless in the context of endemic diseases having reached the stage of endemic stability, which is often the case under traditional husbandry systems in the developing world. In 2013, we wrote a paper titled: “A One Health surveillance and control of brucellosis in developing countries: Moving away from improvisation” [2]. Human brucellosis remains the commonest zoonotic disease worldwide with more than 500 000 new cases annually. The disease is caused by various *Brucella* species, which mainly infect cattle, swine, goats, sheep. Humans generally acquire the infection through direct contact with infected animals, by eating or drinking contaminated animal products, or by inhaling airborne agents. The most rational approach for preventing human brucellosis is the control and elimination of the infection in animals and the pasteurization of milk [3]. The aim of the aforementioned publication was to highlighting knowledge gaps and misunderstandings about the biology of *Brucella* infections. Understanding the biology of *Brucella* infections and the transmission patterns at the wildlife/livestock/human interface is of paramount importance before implementing any control or eradication program in animals, even more so should interventions be justified within One Health (OH) label. Indeed, in addition to calling for transdisciplinary collaboration, and in contrast to the standard common (veterinary) public health paradigm, which is anthropocentric, the OH paradigm aims to promote the well-being of animals and natural resourse conservation and management [4]. Unfortunately, many authors seem to view OH as nothing more than a call for interdisciplinary collaboration, the latter often being only informal. OH is much more than cost saving by sharing transport, software, communication systems and laboratory infrastructure: it requires core competencies [5]. A new curriculum developing transdisciplinary core OH competencies, beyond the discipline-specific area of expertise, is therefore needed in order to design and implement true OH approaches. Unfortunately, up to this day, education remains often segregated between human health, animal health and environmental health (discipline-specific silos) [6].

The aim of this opinion paper is to review the OH approach to brucellosis interventions implemented in industrialized and in low and middle income countries (LMICs) and to question whether, standard-culling practices are efficient and ethically sound OH measures, both for brucellosis control and more generally in health interventions.

## OH justification of brucellosis in industrialized countries

### Veterinary public health or one health?

One of the hallmark of OH interventions is cost-effectiveness and economic benefits to human society and the agricultural sector [7]. Before OH gained momentum, cost-effectiveness and economic benefit had been the primary rationale for the implementation of brucellosis eradication programs in industrialized countries. In the European Union (EU), such programs have been in place more than five decades (Council Directive 64/432/EEC). Yet, to this day, eradication of brucellosis in cattle and small ruminants has not been achieved in some regions of some Member States (MS) [8]. From an economic point of view, achieving and maintaining the “brucellosis free” status is of the utmost importance. If this status is lost, national and international veterinary regulations impose restrictions and bans on animal movements and trade, culling of animals both (infected onesand those at risk of infection), additional testing, and other logistic and administrative measures [9]. In such situations, brucellosis is considered almost exclusively as an economic disease of livestock, whose management is the responsibility of national Veterinary Services. Interventions focusing on acquiring and maintaining the “brucellosis-free” status are neither viewed, nor implemented in a OH perspective, only as veterinary public health measures and only in some MS [10].

### The economic dimension

Except for very few studies, there is hardly any scientific bibliography on methods to estimate costs of interventions and perform economic analysis in veterinary public health [10]. This is rather surprising within the EU, as MS must provide cost projections when applying for EU co-financial contributions to control, monitor or eradicate animal diseases (Council Decision 2009/470/EC). Recently, a cost description of eradication programs of bovine tuberculosis, brucellosis and leucosis for the region of Lazio, Italy during the period 2007–2011 has been published [10]. The total cost for the eradication programs for these three diseases, adjusted for inflation to 1 January 2016, was estimated at about 19 million euro (about 6 million euro for brucellosis) for an average number of 236.262 cattle heads and 12.538 cattle herds. It is worth noting that the study showed that costs decreased by 50% after the acquisition of the “disease free” status. The underlying reason of this dramatic reduction is that the testing frequency and the target population that must be tested according to regulations can be reduced with the acquisition of the “disease free” status. Importantly, the total cost did not include costs associated with movement restrictions and additional losses borne by farmers, except those for the culling of animals, as they were not considered not to be the responsibility of the competent authority. This is one of the main reasons explaining why there is often strong reluctance and opposition to such programs from the producers. Nevertheless, documenting that costs linked to the eradication program are reduced by more or less 50% is important to convincing policy makers that in a period of austerity, reduction of public services and increasing budget constraints, it would be counterproductive to jeopardize the attainment of brucellosis free status. On the contrary, the allocation of additional resources when acquiring the brucellosis free status is in reach, is a sound and cost-effective veterinary public health measure [10].

## OH justification of brucellosis in LMICs

### Moving away from serology to *Brucella* isolation, identification and characterization

In a recent paper, the main considerations for prioritizing and planning a surveillance system for endemic zoonotic diseases in LMICs were described and discussed. The aim of such a system is to monitoring for case detection and disease prevalence estimation. Importantly, appropriate intervention capacity and sufficient resources to implement control measures must be available [11]. The vast majority of the brucellosis surveillance systems in LMICs are based almost exclusively on serological testing [12]. When brucellosis reaches endemic stability [13], serological inquiries do not need to be repeated because, per definition, the epidemiological situation is stable. In addition, in such situations very little (if any) intervention is reported to be implemented. OH approaches should aim to produce positive outcomes that are resilient, productive, and endure over time [14]. Thus, one is entitled to asking the following: is monitoring of brucellosis by serology (still) a OH priority in LMICs? To answer this question, it is important to be first reminded that an often forgotten shortcoming of brucellosis serology is the impossibility to infer which (smooth) *Brucella* spp. induced antibodies in the host [15]. In this respect, mixed farming and especially keeping small ruminants along with cattle, a common practice in LMICs is a recognized risk factor. However, a central question that has to be answered is whether cattle and small ruminants are infected with *Brucella melitensis* or with *Brucella abortus* or with both *Brucella* species [2]. The situation seems to be very different between West and East Africa. Indeed, in West Africa, only *B. abortus* has been isolated from cattle and from small ruminants [16], whereas in East Africa, cattle are infected with *B. abortus*, as indicated by studies in Zimbabwe [17], Tanzania [18] and Uganda [19, 20], or with both *Brucella* species, as recently described in Kenya [20]. Additionally, the recent occurrence of *B. suis* infection along with *B. abortus* and *B. melitensis* infections in cattle in Egypt highlights the urgent need to study brucellosis in pigs in Africa [21]. To date no documented report on its isolation in pigs in Sub-Saharan Africa is available in the international literature. Further, it is worth mentioning that *B. abortus* and *B. melitensis* have been detected in camels, a very important livestock species in East Africa, North Africa and the Middle East [22]. The identification of *Brucella* spp. from infected animals is critical if specific interventions have to be implemented. Indeed, besides providing material to performing molecular epidemiology and documenting transmission patterns, it is important to recognize that *B. melitensis* infection in cattle has only been reported when a source of *B. melitensis* is found in its preferential hosts, i.e., small ruminants such as in Spain [23] and France [24], when bovine brucellosis (*B. abortus*) was almost eradicated. Likewise *B. abortus* infection in small ruminants is reported when a source of *B. abortus* is found in its preferential host, i.e., cattle [16, 25]. This suggests that *B. melitensis* infection in cattle and *B. abortus* infection in small ruminants are spill over infections under traditional husbandry systems. A consequence of this is that if vaccination is considered a sound intervention, it should first of all be implemented in reservoir hosts i.e. cattle for *B. abortus* and small ruminants for *B. melitensis*, not in spill over hosts, even more so in countries where resources are scarce [2]. Additionally, it is not known if camels can sustain a *B. melitensis* or a *B. abortus* infection without a constant influx of bacteria from their true reservoir species [22]. It is important to document whether camels are more victims (spillover hosts) than vectors (reservoir hosts) before considering vaccination, notwithstanding the fact that there is no registered brucellosis vaccine for camels.

### Urbanization

According to the United Nations, today, 54% of the world’s population lives in urban areas, a proportion that is expected to increase to 66% by 2050. Projections show that urbanization combined with the overall growth of the world’s population could add another 2.5 billion people to urban populations by 2050 [26]. Livestock farming in urban and peri-urban areas is increasing in LMICs in parallel to the rapid urbanization. It represents an important source of animal food products to the increasing urban populations and a recent study in Uganda suggested that there may be variations in risk factors for *Brucella* transmission in cattle related to the geographical location of the animal i.e. rural or urban [27]. To the contrary of what is seen in the vast majority of traditional husbandry systems, brucellosis in urban and peri-urban farming systems is likely not to have reached endemic stability, and in this case monitoring and estimating prevalence may be required. In some African countries, in the Middle East and in Asia, livestock farming in urban and peri-urban environments are often characterized by low biosecurity where different animal species are kept in close proximity to humans [28, 29]. In these environments, ewes, does and cows are unable to express their natural seclusive behavior at parturition. Consequently, the *B. melitensis* infectious pressure for cattle may be enhanced. A possible consequence of this is that some *B. melitensis* strains may cross the interspecies barrier and may be sustainably transmitted among cattle, without the constant influx of *B. melitensis* from small ruminants [30]. It is however important to note that this remains to be demonstrated before advocating the vaccination of cattle against *B. melitensis*. Altogether, this re-enforces the fact that a tailored approach has to be considered when designing control measures for prevention of transmission of brucellosis in livestock farming in urban and peri-urban areas in order to protect both livestock and people from infection [29].

Recently, the relative contributions of food to the burden of disease due to selected foodborne hazards has been estimated by the World Health Organization (WHO) through a structured expert elicitation [31]. For *Brucella* spp., there is a clear pattern that the foodborne proportion is more important in the industialized countries and LMICs. Direct animal contact was considered equally or more important than foodborne transmission in developing world. The development of urban and peri-urban farming is likely to enhance this trend. As a result, brucellosis veterinary public health measures targeting direct contact with animals, more than measures targeting milk and milk products may have to be prioritized in the future.

Urbanization also creates new habitats for wildlife and thus dynamics for disease emergence at the wildlife/livestock/human interface. Urbanization provides very different living conditions to new residents and living in slums is known to be a health hazard because of poor housing, high-density populations, lack of fresh water and weak hygiene practices and sanitation facilities. This urban environment has proven to be favorable for rodent populations and vector proliferation, enhancing the risk for zoonotic infectious and parasitic diseases. Early detection and identification of these new threats as well as deciphering the routes of transmission at the interface will be important in order to implement sound mitigation interventions in the near future [29].

## One health and wildlife brucellosis

Besides *Brucella* spill-over from livestock to wildlife species, *B suis* biovars 2 and 4 have reservoir hosts in wildlife: wild boars (*Sus scrofa*) and reindeer (*Tarandus tarandus*), respectively. Recently, new species of *Brucella* have been described and validly published in marine mammals, rodents, foxes, frogs and monkeys [32].

Nowadays, it is recognized that four free-ranging wildlife species are currently self-sustaining reservoirs of *B. abortus* or *B. melitensis* and are potential sources of livestock infections (spill-back). Bison (*Bison bison*) and elk (*Cervus canadensis*) in the Greater Yellowstone Ecosystem in the USA, and African buffalo (*Syncerus caffer*) in South-East Africa sustain *B. abortus* [33, 34], whereas the Alpine ibex (*Capra ibex*) in the French Alps, sustains *B. melitensis* [35]*.* The main concern is related to management to avoidi spill-back from wildlife to livestock. In the case of *B. abortus* in elk and bison spill-back have been documented and management practices based on spatio-temporal segregation and culling of animals crossing the border of the Greater Yellowstone Ecosystem are implemented. Management practices, such as winter feeding, are still a matter of heavy debates between different interest groups [34]. In South Africa, brucellosis in buffalo is monitored but no spill-back to livestock has been documented and no brucellosis specific management practices are implemented Simpson et al., submitted manuscript). For the population of the Alpine ibex of the Bargy range in the French Alps, spill-back to cattle and transmission from cattle to people via the consummation of infected cheese, has been reported, although not demonstrated [35]. In 2013 and 2015, the French competent authorities allowed the culling of animals that are protected by international conventions, which generated strong opposition and concern. Nowadays, different management options are considered, among which vaccination of the Alpine ibex. These different intervention options highlight the difficulties to conciliate animal health and environment health approaches and question the culling of wildlife aiming at reducing the risk of transmission to livestock [36].

## Beyond brucellosis, the ethical dimension of culling to control zoonotic diseases

Difficulties managing the ongoing H5N1 high pathogenic avian influenza (HPAI) have propelled OH approaches on the global health agenda with the endorsement of OH approaches by international organizations (FAO-OIE-WHO, 2010; World Bank, 2010) firstly to guiding their collaborative efforts to control HPAI. HPAI influenza almost always arises from low pathogenic avian influenza (LPAI) subtypes H5 and H7. Thus, the emergence of LPAI viruses of these two subtypes in poultry has now become the trigger for aggressive test-and-slaughter policies [37].

Among the 3 basic objectives of OH, i.e., human health, environment health and animal health, a legitimate debate arises as to whether culling of livestock and/or wildlife species is an ethically justified OH approach [38]. Culling or “stamping-out” remains the major strategy for epidemic diseases like foot-and-mouth disease or endemic diseases like bovine leucosis, which are not zoonotic diseases. The rationale is that culling is aiming at eliminating or reducing reservoir populations, and as a result, decreasing or eventually stopping transmission of pathogens to naïve hosts. Some scientists argue that the evidence documenting the epidemiological efficacy and cost-effectiveness of culling as a sustainable solution to many zoonotic and epidemic diseases remains inconclusive [38]. Objections to culling rest on whether the killing of animals is acceptable, should alternative approaches to infectious disease control be available. Bovine brucellosis has been successfully eradicated in many industrialized countries by applying a test-and-slaughter policy (after banning vaccination). However, this approach is culturally not acceptable in many LMICs, even if compensation schemes were to be implemented. Indeed, livestock represents much more than monetary compensation, notwithstanding the fact that in countries like India, the slaughtering of cattle is not allowed for religious reasons. Nonetheless, these endemic diseases are to a certain degree controlled in many LMICs, in the absence of culling [2]. Exploring traditional [39] and other methods to control diseases such as ethnoveterinary medicine [40], combining traditional knowledge with new technologies like meteorological and ecological information to predict and prevent disease outbreaks [41], is ethically and scientifically justified.

## Concluding remarks

The most important contribution of OH is empirically to demonstrate the interdependence of humans, ecosystems and animals in both sickness and health. However, even if we consider animals as morally valuable, we consider them as less valuable than humans, and therefore it is justified to subjecting them to culling for the potential future benefit of future generations of both humans and animals. Conversely, if we conclude that culling is not ethically justified, then even if we prevent animal suffering, culling would remain unacceptable. OH potentially constitutes a paradigm shift in our worldview, compelling us to revisit our perception of the moral status of animals, plants and ecosystems.

The University of Tromsø - the Arctic University of Norway is offering to PhD students the BIO-8603 course on “Philosophy of Science and Ethics”. Within this course, I am giving teaching hours on “Environmental Ethics”. In my teaching, I am referring to Arne Dekke Eide Næss (1912-2009), a Norwegian philosopher who coined the term “deep ecology” in 1973. He distinguished between what he called deep and shallow ecological thinking. In contrast to the prevailing utilitarian pragmatism of western businesses and governments, he advocated that a true understanding of nature would give rise to a point of view that appreciates the value of biological diversity, understanding that each living thing is dependent on the existence of other creatures in the complex web of interrelationships that is the natural world. Plants and animals are internally related. Manipulating them can have unintended and often unwelcome consequences. *Homo sapiens* is but one evolved species among myriads of others with no claim to special privilege. Isn’t this an OH approach from the ecological perspective? In this respect, a recent publication measured interdisciplinarity in OH studies construct in dynamic pathogen transmission models, in the published scientific literature. Publications clustered into three communities: ecologists, veterinarians, and a third on used by population biologists, mathematicians, epidemiologists, and experts in human health. Fortunately, the overlap between these communities increases overtime but some segregation, particularly at the veterinary/ecological research interface, remains [42].

Core transdisciplinary competencies like values and ethics are critically important for guiding OH curriculum development and continuing professional education, as they describe the knowledge, skills, and attitudes required to be effective. Transdisciplinary research is currently poorly financed. A conceptual framework needs to be developed in such research activities from inception to knowledge translation. It should be re-emphasized that transdisciplinary competencies should be developed besides discipline-specific areas of expertise, not to replace them. This is why the understanding of the biology of infectious diseases is and will always remain a pre-requisite for any sound OH approach [30].
